# Histopathological Insights into Primary Localized Cutaneous Amyloidosis: A Case Series

**DOI:** 10.7759/cureus.79603

**Published:** 2025-02-24

**Authors:** Dinisha Einstien, Sarumathy G, Shobini Vishali V M, Prathiba A, Perumal M, Sruthi Pallekonda

**Affiliations:** 1 Pathology, Panimalar Medical College Hospital & Research Institute, Chennai, IND; 2 Dermatology, Panimalar Medical College Hospital & Research Institute, Chennai, IND

**Keywords:** amyloid deposition, congo red staining, histopathology, lichen amyloidosis, macular amyloidosis, primary localized cutaneous amyloidosis

## Abstract

Background

Primary Localized Cutaneous Amyloidosis (PLCA) is a rare disorder characterized by amyloid deposition in the skin without systemic involvement. It includes lichen amyloidosis (LA), macular amyloidosis (MA), and biphasic amyloidosis. The pathogenesis is linked to chronic friction, keratinocyte damage, and in some cases, genetic predisposition. Histopathological examination remains the gold standard for diagnosis. Here, we present a series of nine cases of PLCA.

Objective

This case series aims to establish a correlation between the clinical manifestations of PLCA and the histopathological findings, including special stains, to improve diagnostic accuracy. Identifying these correlations is vital for differentiating PLCA subtypes, guiding treatment strategies, and understanding the underlying pathogenic mechanisms.

Methods

A retrospective analysis was conducted at Panimalar Medical College and Hospital, Chennai, India, reviewing nine biopsy-confirmed PLCA cases from January 2023 to December 2024. Clinical data, histopathological features, and staining characteristics (hematoxylin & eosin, Congo red, and crystal violet) were analyzed.

Results

Out of nine cases, LA was the predominant subtype (seven cases), followed by MA (two cases). Female predominance was observed (male-to-female ratio was 1:2), with an age range of 39-65 years. The upper limbs were the most frequently affected site. Pruritus was exclusively seen in LA, while hyperpigmentation was present in all cases. Histopathological analysis revealed amyloid deposits in the papillary dermis, epidermal hyperplasia, and inflammatory infiltration in LA. Congo red staining demonstrated characteristic apple-green birefringence under polarized light, confirming amyloid deposition.

Conclusion

The study reinforces the clinical and histopathological distinctiveness of PLCA subtypes. LA is strongly associated with chronic friction and pruritus, whereas MA presents with asymptomatic hyperpigmentation. Congo red and crystal violet stains remain indispensable for diagnosis. Despite advances in histopathology, therapeutic options remain limited, necessitating further research into targeted molecular treatments and genetic predispositions.

## Introduction

Amyloidosis comprises a group of disorders characterized by extracellular deposition of insoluble fibrillar aggregates due to protein misfolding. Initially described by Rudolf Virchow in 1854, amyloid deposits were later identified as proteinaceous rather than polysaccharide-based [1]. These deposits primarily consist of fibrillar proteins in a beta-pleated sheet conformation, along with non-fibrillar components such as serum amyloid P, apolipoprotein E, and glycosaminoglycans, contributing to structural stability and pathogenesis [2].

Amyloid deposition occurs due to genetic mutations or misfolding of normal proteins, overwhelming cellular clearance mechanisms [3]. Currently, 36 amyloidogenic proteins are known to cause amyloidosis in humans [4]. Based on distribution, amyloidosis is classified as systemic or localized. Systemic amyloidosis affects multiple organs, with AL amyloidosis (light chain Amyloidosis, derived from immunoglobulin light chains) being the most prevalent form [5]. Hereditary systemic amyloidosis is associated with mutations in transthyretin (ATTRv), fibrinogen (AFib), apolipoproteins (ApoAI, ApoAII, ApoC-II, ApoC-III), gelsolin (AGel), and lysozyme (ALys) [6].

Skin involvement can be primary (localized) or secondary to systemic amyloidosis. Systemic amyloidosis with cutaneous manifestations is most commonly observed in AL and AA (serum amyloid A) amyloidosis [7]. Primary localized cutaneous amyloidosis (PLCA) occurs without systemic involvement and includes lichen amyloidosis (LA), macular amyloidosis (MA), and biphasic amyloidosis [8]. LA presents as pruritic, hyperkeratotic papules on extensor surfaces, linked to keratinocyte damage from chronic scratching. MA features hyperpigmented, pruritic macules in a reticulated or rippled pattern, commonly on the trunk and extremities [9]. Biphasic amyloidosis represents coexistence or transformation of MA into LA, often induced by persistent scratching [10]. Nodular localized cutaneous amyloidosis, a rare form, involves AL-type amyloid deposition [11]. While most cases of LA are sporadic, up to 10% of cases are inherited in an autosomal dominant pattern with variable penetrance. Familial PLCA is linked to oncostatin M-specific receptor (*OSMR*) gene mutations, predisposing individuals to LA and MA [12].

Histologically, LA is characterized by homogenous amyloid deposits, typically confined to the papillary dermis, and visible with stains like Congo red or crystal violet. Additional histological features may include irregular acanthosis, orthohyperkeratosis, and colloid bodies. It's important to differentiate LA from several other conditions, including hypertrophic lichen planus, lichen simplex chronicus, and lichen striatus. Other mimickers include prurigo nodularis, pretibial myxedema, papular amyloidosis, and elephantiasis nostras verrucosa. When considering MA, the differential diagnosis should include post-inflammatory hyperpigmentation, erythema dyschromicum perstans, drug-induced pigmentation, frictional melanosis, and notalgia paraesthetica [9].

## Materials and methods

This study was conducted at Panimalar Medical College and Hospital, Chennai, Tamil Nadu, India, with clinical and histopathological data retrieved from the Department of Pathology archives. Cases were identified based on a retrospective review of skin biopsies reported from January 2023 to December 2024. All cases with informed consent and histologically confirmed diagnosis of PLCA were included in the study. Cases with systemic involvement and those lacking confirmation of diagnosis with special stains were excluded. The cases were evaluated based on age, sex, site of lesions, symptoms, and examination findings.

This is a case series of nine patients who presented to the Dermatology outpatient department with pruritic, hyperpigmented patches, plaques, and papules and were clinically diagnosed with cutaneous amyloidosis. Systemic involvement was ruled out in all cases. To confirm the diagnosis, punch biopsy samples were obtained and processed using standard histopathological techniques.

Tissue sections were stained with hematoxylin & eosin (H&E), revealing amorphous, eosinophilic amyloid deposits. Histochemical staining was done with crystal violet for demonstrating metachromasia and Congo red highlighting amyloid deposits with a brick-red color and exhibiting apple-green birefringence, under polarized light microscopy, thereby confirming amyloid deposition. As this was a descriptive study, no statistical analysis was done.

## Results

This study analyzed the clinical and histopathological characteristics of nine patients diagnosed with cutaneous amyloidosis. The majority (seven cases) presented with LA, while two cases had MA. Female predominance was observed with a male-to-female ratio of 1:2, three males and six females), with an age range of 39-65 years. None of the cases had any co-morbidities. Systemic involvement was ruled out in all cases. The upper limbs (four cases) were the most commonly affected site, followed by the lower limbs (three cases) and upper back (two cases). 

**Figure 1:**
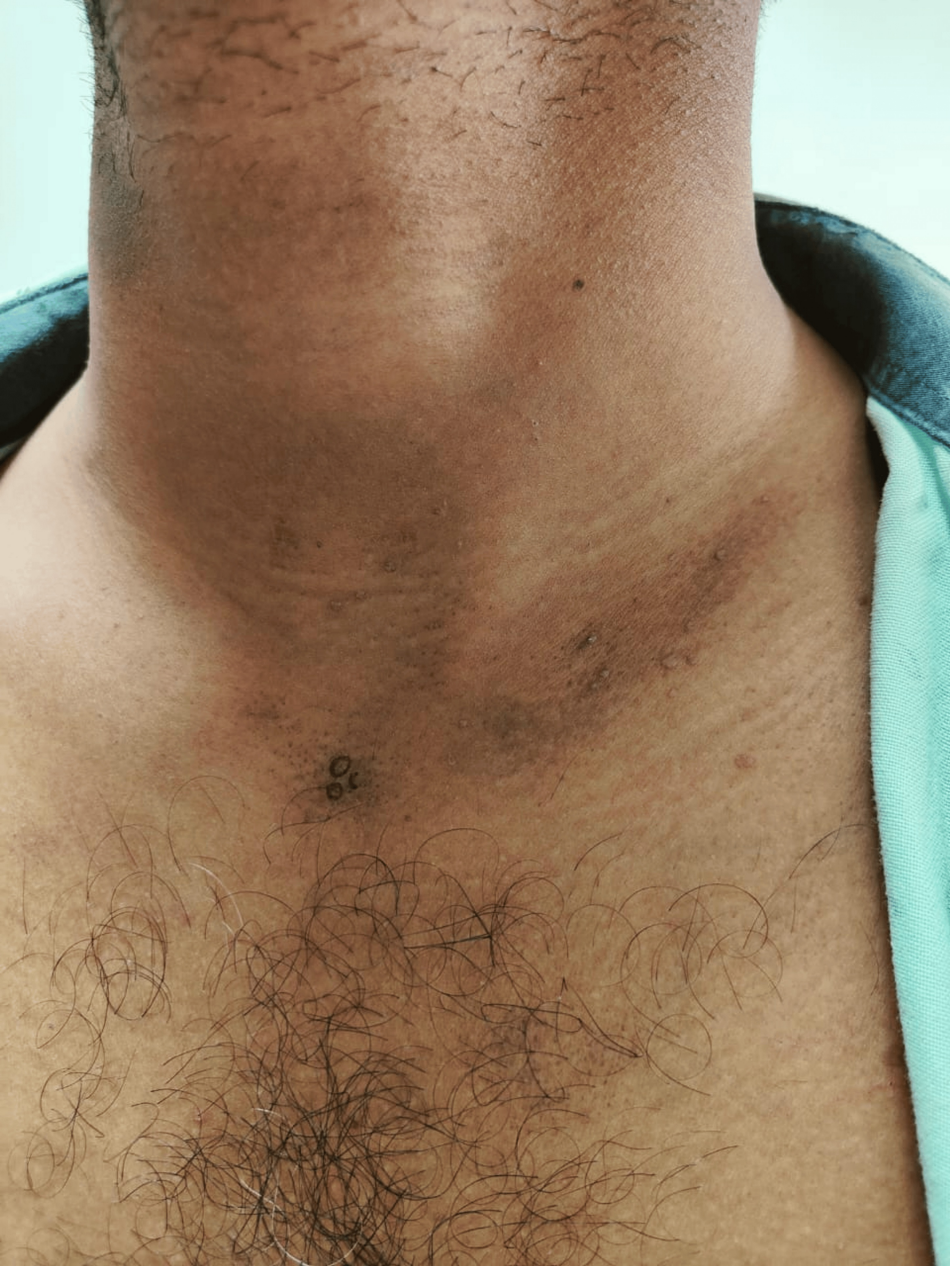
Hyperpigmented lesions of PLCA PLCA: primary localized cutaneous amyloidosis

**Figure 2:**
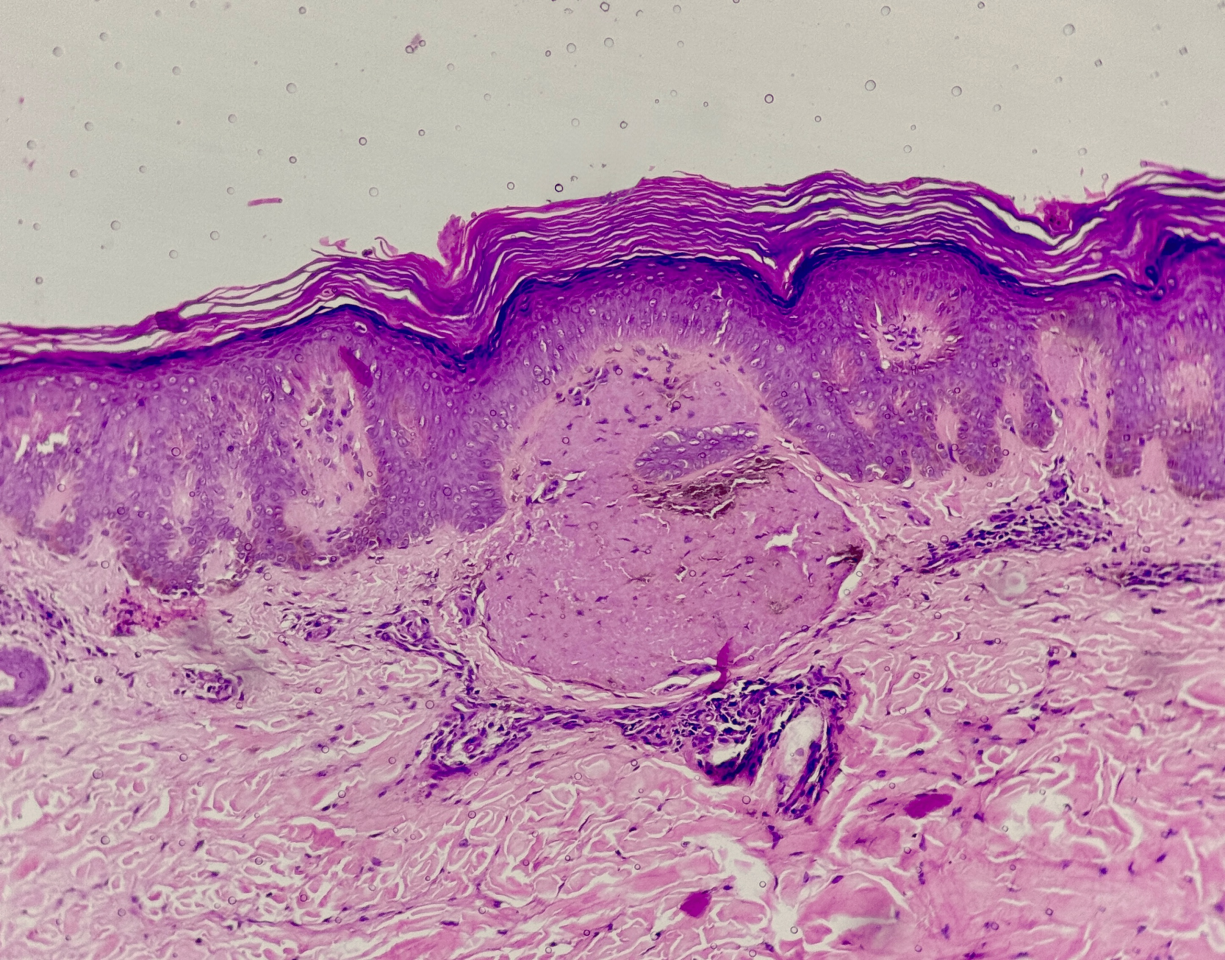
Amyloid deposit in the papillary dermis (hematoxylin & eosin stain, 100x)

**Figure 3:**
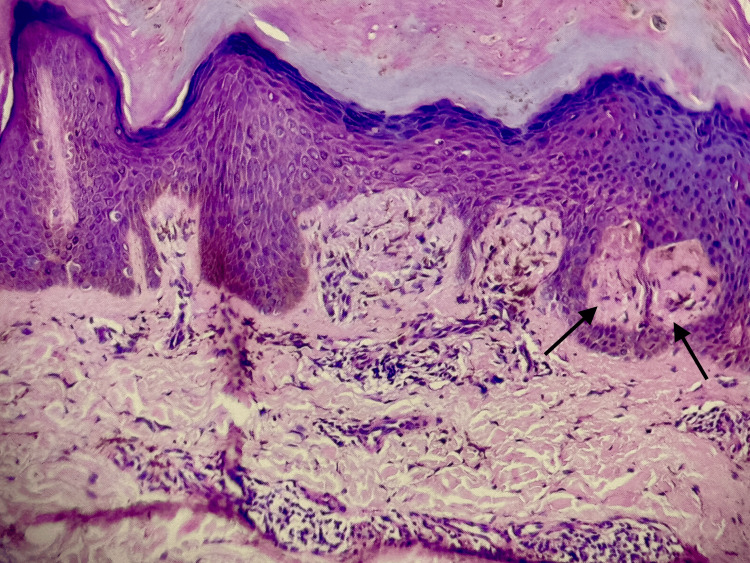
Epidermal hyperplasia with hyperkeratosis, mild acanthosis, and inflammatory infiltration with elongation of rete ridges observed in lichen amyloidosis. The amyloid deposits are marked with arrows (hematoxylin & eosin stain, 400x)

**Figure 4:**
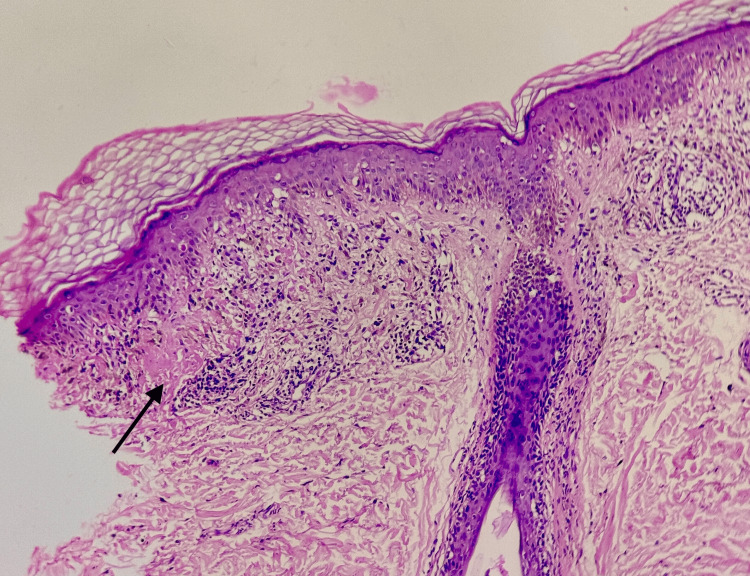
Amyloid deposition (arrow) with inflammatory infiltrates and no marked epidermal changes in macular amyloidosis (hematoxylin & eosin stain, 100x)

**Figure 5:**
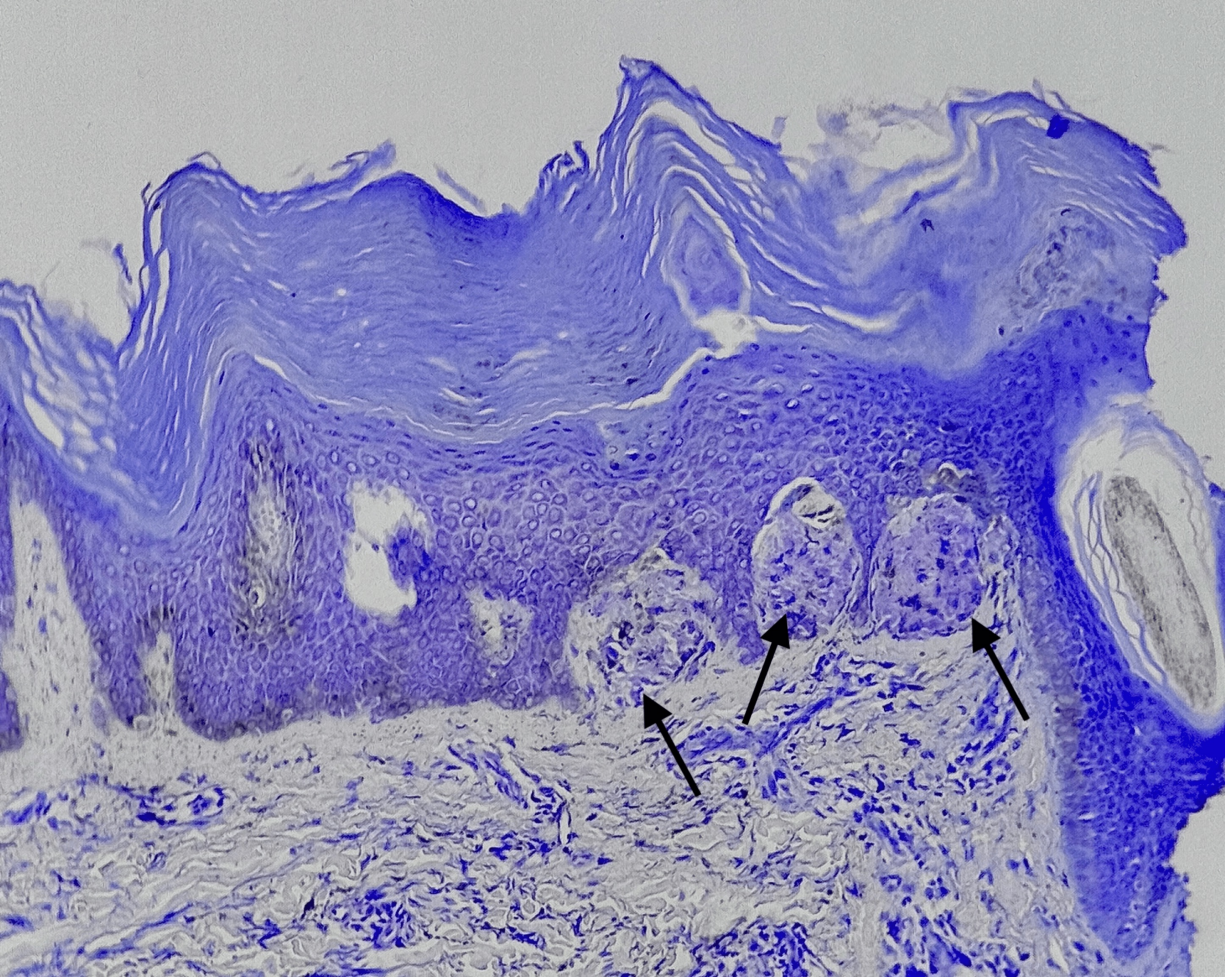
Amyloid deposits as highlighted by crystal violet stain (100x)

**Figure 6:**
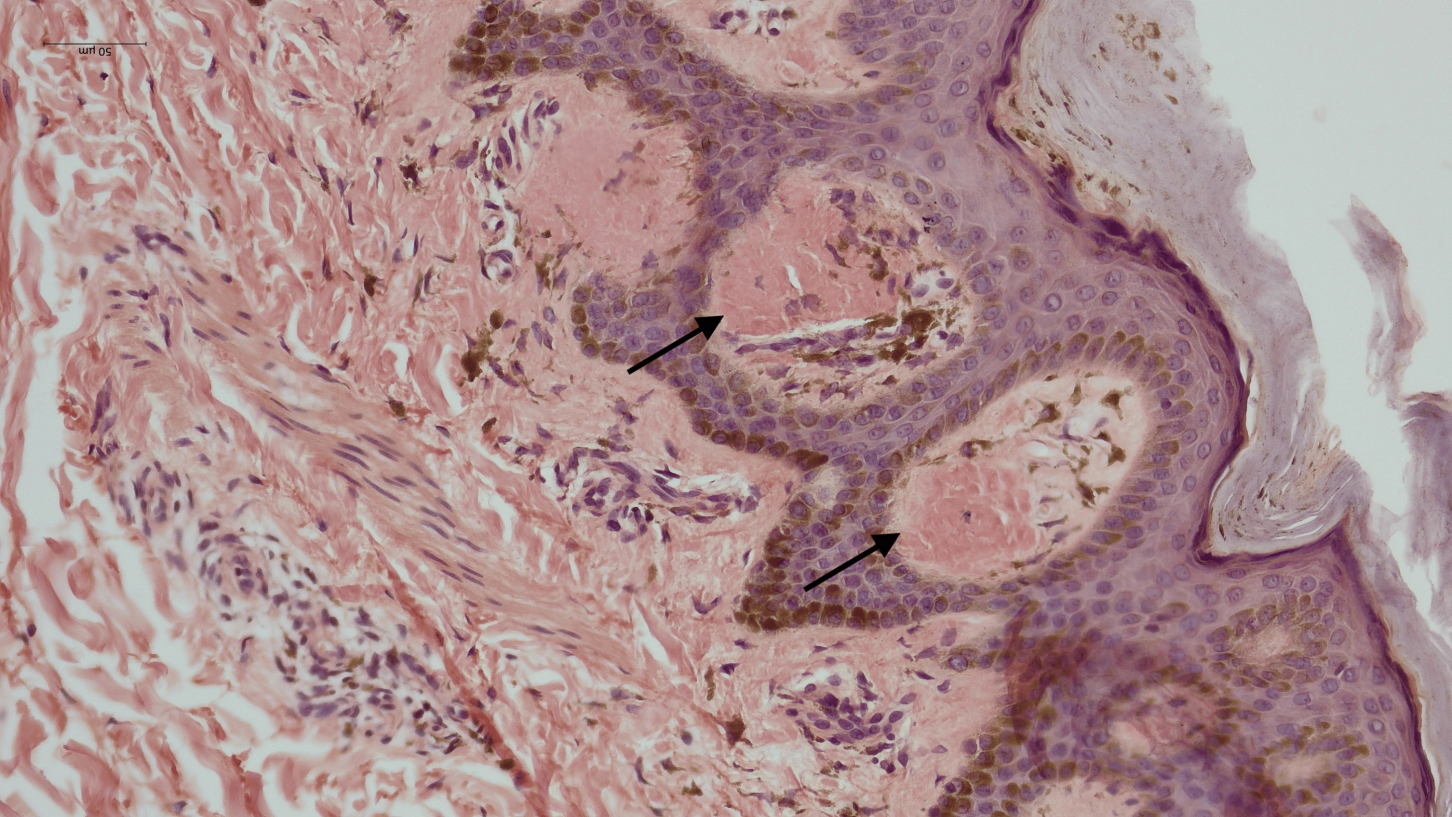
Amyloid deposits in the papillary dermis as highlighted by Congo red stain (400x)

**Figure 7:**
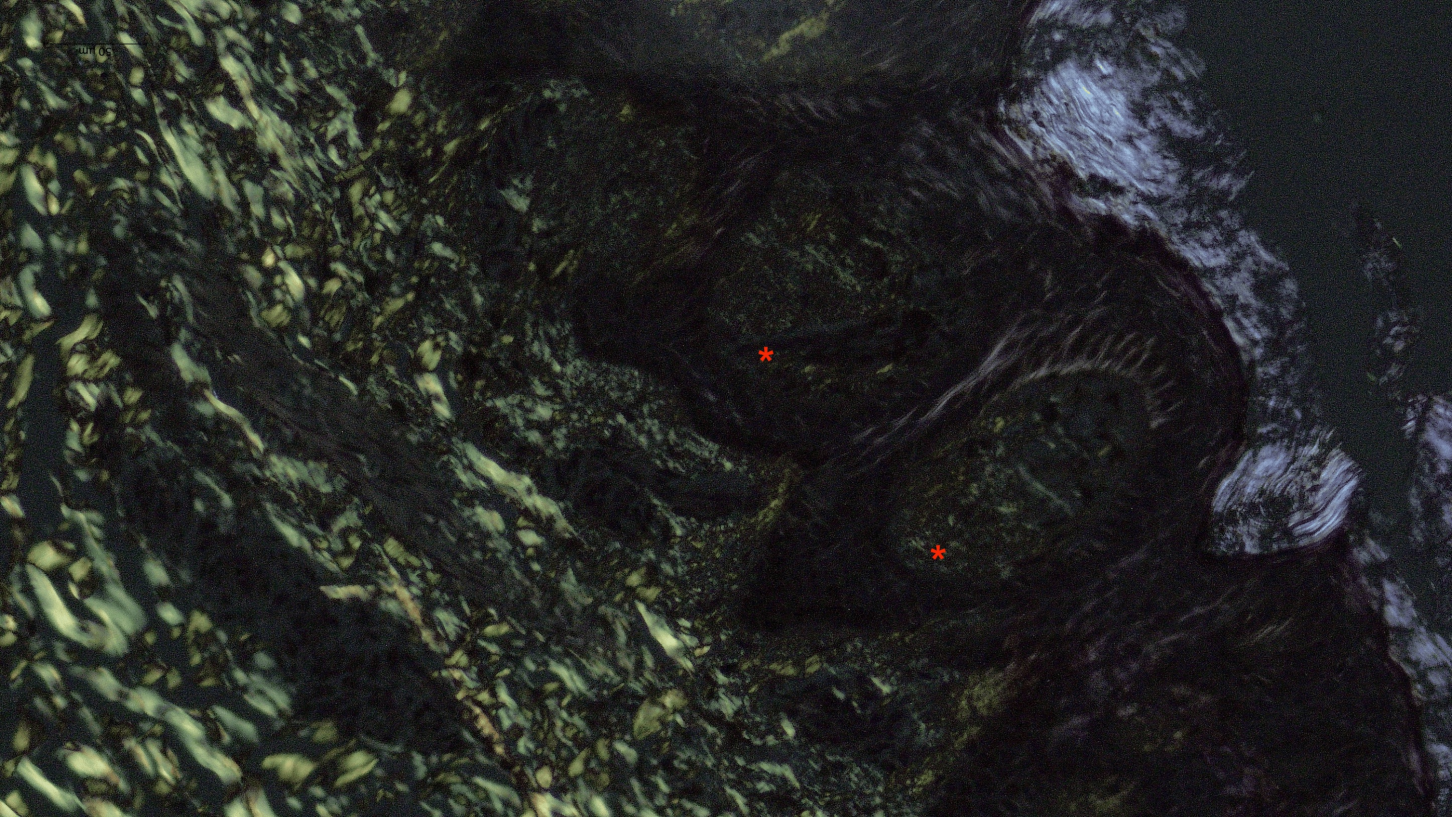
Apple green birefringence of Congo red-stained amyloid (red asterix) observed under polarizing microscope, 400x.

Hyperpigmentation (Figure 1) was the primary symptom in all cases, with pruritus exclusively observed in all cases of LA. The histopathological features observed are tabulated in Table 1. Histopathological examination confirmed amyloid deposits in the papillary dermis (Figure 2) in all cases, along with epidermal hyperplasia, inflammatory infiltration, and rete ridge elongation in LA (Figure 3). The epidermal changes were not marked in cases of MA (Figure 4). Crystal violet (Figure 5) and Congo red (Figure 6) stains confirmed amyloid presence in all nine cases. The characteristic apple-green birefringence with Congo red staining under a polarizing microscope (Figure 7) was seen in all cases.

**Table 1:** Histopathological findings observed in primary localised cutaneous amyloidosis (N=9)

Histopathological features	Lichen Amyloidosis (n=7)	Macular Amyloidosis (n=2)	Total (n=9)
Epidermal Changes			
Hyperplastic squamous epithelium	5	1	6
Focal parakeratosis	1	0	1
Acanthosis	1	0	1
Hypergranulosis	1	0	1
Dermal Changes			
Amyloid deposits (papillary dermis)	Present in all cases	Present in all cases	9
Inflammatory infiltration	Perivascular (n=5), Nodular (n=1)	Minimal to absent	-
Thickened & elongated rete ridges	2	0	2
Enlarged nerve bundles (deep dermis)	1	0	1
Pigment incontinence	1	0	1
Special Stains for Amyloid			
Crystal Violet	Positive in all cases	Positive in all cases	9
Congo Red	Positive in all cases	Positive in all cases	9
Congo Red (Polarized Light)	Apple-green birefringence in all cases	Apple-green birefringence in all cases	9

## Discussion

This study aimed to correlate the clinical presentations of PLCA with histopathological findings, shedding light on its diverse manifestations and underlying pathology. Nine patients with cutaneous amyloidosis were evaluated clinically and histopathologically. The findings of this study align with the established subtypes of PLCA: LA (seven cases) and MA (two cases), consistent with previous studies that have highlighted the predominance of LA in cutaneous amyloidosis cases [13,14]. The age range of 39-65 years with female preponderance is similar to the observations in other cohorts of cutaneous amyloidosis patients [13-22]. In a recent retrospective monocentric study by Guillet et al., investigating the epidemiologic characteristics of PLCA, female individuals were found to be more likely to develop PLCA, and LA was the most frequent subtype [14].

LA predominantly presented as pruritic hyperpigmented papules coalescing into plaques, primarily involving the lower limbs. This is similar to earlier studies indicating a predilection for extensor surfaces of the legs and the significant role of chronic scratching or friction in its pathogenesis​​ [13-15]. MA, in contrast, presented with non-pruritic, hyperpigmented macules in a rippled pattern, most commonly on the trunk and upper limbs, which is consistent with the clinical description by Rasi et al. [16]. In both forms, hyperpigmentation was a prominent feature, as reported by Somani et al. [17]. The pruritus observed in LA, but absent in MA, further supports the hypothesis that scratching and chronic pruritus might damage keratinocytes and trigger amyloid deposition in the dermis, particularly in LA. However, Ramírez-Santos et al. observed cases of cutaneous amyloidosis not associated with scratching, and chronic irritation was not the causal factor [18].

Histopathological analysis remains the gold standard for diagnosing PLCA. In this study, H&E staining revealed characteristic amorphous, eosinophilic deposits in the papillary dermis, characteristic finding of cutaneous amyloidosis [13-22]. It was further confirmed by crystal violet and Congo red staining, with Congo red exhibiting apple-green birefringence under polarized light. The consistent positivity of these stains across all cases is consistent with the results of prior studies emphasizing the diagnostic utility of histochemical stains in confirming amyloid deposition​ [20-22]. Additional stains like Thioflavin T, which is selective for amyloid fibrils [15], and immunohistochemistry with CK5/6 can further aid in the diagnosis of amyloid deposition. Sinha et al. reported 100% sensitivity with Congo red and with CK5/6, suggesting CK5/6 as an alternative tool to Congo red stain in the diagnosis of primary cutaneous amyloidosis [20]. 

The epidermal changes observed in both LA and MA include hyperplasia, acanthosis, and hypergranulosis, as observed by Mehrotra et al. [21]. These epidermal alterations are characteristic of chronic inflammatory skin conditions, where persistent irritation and inflammation lead to skin remodeling and thickening. The inflammatory infiltrates observed in LA cases in our study predominantly comprised perivascular lymphocytes and plasma cells, a feature that has been documented by Vijaya et al. too [22]. The histopathological changes of MA were subtler, characterized by minimal to absent inflammation and a lack of prominent epidermal alterations, a finding consistent with its clinical presentation as non-pruritic, rippled hyperpigmentation [16]. The presence of enlarged nerve bundles in some cases of LA raises intriguing questions about the relationship between neural changes and pruritus. Tey et al. suggested that pruritus in PLCA is likely associated with hypersensitivity of cutaneous nerve fibers, which may be related to an increased expression of epidermal IL-31 receptors [23]. The presence of pigment incontinence in one case of LA suggests that melanocytes may be damaged due to chronic scratching.

Cutaneous amyloid deposits are thought to be derived from degenerated keratin peptides of apoptotic keratinocytes transformed into amyloid fibrils by dermal macrophages and fibroblasts. A working hypothesis is that the epidermal trauma induced by long-term scratching and rubbing seen in associated chronic diseases results in keratinocyte degradation and the formation of amyloid [19]. Familial cases of PLCA, particularly those linked to mutations in the *OSMR* gene, suggest a genetic predisposition in certain individuals. Although our study did not evaluate familial patterns, the autosomal dominant inheritance pattern reported in other studies implies the importance of considering genetic testing in recurrent or familial cases of PLCA [12,24]. The rare phenomenon of amyloid deposition associated with neoplasms, such as basal cell carcinoma (BCC) and adnexal tumors, also warrants attention. Secondary cutaneous amyloidosis has been reported in mycosis fungoides, where chronic inflammation and keratinocyte turnover contribute to localized amyloid formation. It has also been associated with Bowen's Disease and Bowenoid Papulosis [25-27]. Cases initially suspected as BCC and later identified as PLCA upon histopathological examination have also been reported [28].

Despite advancements in the diagnostic techniques for PLCA, therapeutic options remain limited and largely symptomatic. Pruritus, the most troubling symptom in LA, poses a significant challenge to quality of life. Current treatments, including topical steroids, keratolytics, and systemic therapies like retinoids, aim to reduce inflammation and pruritus but have variable efficacy. Novel approaches targeting the molecular mechanisms of amyloid formation, such as inhibitors of protein aggregation, hold promise but require further clinical validation​.

The primary limitation of this study is its small sample size, reflecting the rarity of the condition. Since this study was conducted at a single center, its findings may have limited generalizability. Larger multicenter studies are needed to better understand the genetic, environmental, and immunological factors contributing to PLCA. Other limitations include the retrospective design, which may introduce selection bias and recall bias limiting generalizability. However, every effort has been made to ensure the accuracy of the details provided. Immunohistochemistry was not done in this study. Integrating advanced diagnostic modalities like immunohistochemistry and direct immunofluorescence could provide deeper insights into the composition and origin of amyloid deposits.

## Conclusions

The findings from this study reinforce the clinical and histopathological characteristics of LA and MA as distinct forms of cutaneous amyloidosis. The clinical manifestations, including pruritus in LA and hyperpigmentation in both LA and MA, were supported by histopathological findings of amyloid deposition in the dermis, with the application of specialized stains ensuring accurate diagnosis. The study highlights the importance of thorough clinical examination and histopathological evaluation for the correct diagnosis of cutaneous amyloidosis. More multicenter studies are required to understand the genetic, interpersonal, environmental, and cultural aspects of the pathogenesis of PLCA.
